# *Plasmodium matutinum* Transmitted by *Culex pipiens* as a Cause of Avian Malaria in Captive African Penguins (*Spheniscus demersus*) in Italy

**DOI:** 10.3389/fvets.2021.621974

**Published:** 2021-03-16

**Authors:** Manuela Iurescia, Federico Romiti, Cristiano Cocumelli, Elena Lavinia Diaconu, Fiorentino Stravino, Roberta Onorati, Patricia Alba, Klaus Gunther Friedrich, Flavio Maggi, Adele Magliano, Arianna Ermenegildi, Virginia Carfora, Andrea Caprioli, Claudio De Liberato, Antonio Battisti

**Affiliations:** ^1^General Diagnostic Department, Istituto Zooprofilattico Sperimentale del Lazio e della Toscana “M. Aleandri”, Rome, Italy; ^2^Fondazione Bioparco, Rome, Italy; ^3^Zoomarine Acquatic Park, Torvaianica, Rome, Italy

**Keywords:** avian malaria, *Plasmodium* spp., *Plasmodium matutinum*, *Plasmodium vaughani*, mosquitoes, mortality, penguins, zoo

## Abstract

Avian malaria is a parasitic disease of birds caused by protozoa belonging to the genus *Plasmodium*, within the order Haemosporida. Penguins are considered particularly susceptible, and outbreaks in captive populations can lead to high mortality. We used a multidisciplinary approach to investigate the death due to avian malaria, occurred between 2015 and 2019, in eight African penguins (*Spheniscus demersus*) kept in two Italian zoos located in central Italy, and situated about 30 km apart. We also provided information about the presence and circulation of *Plasmodium* spp. in mosquitoes in central Italy by sampling mosquitoes in both zoos where penguin mortalities occurred. In the eight dead penguins, gross and histopathological lesions were consistent with those previously observed by other authors in avian malaria outbreaks. Organs from dead penguins and mosquitoes collected in both zoos were tested for avian malaria parasites by using a PCR assay targeting the partial mitochondrial conserved region of the *cytochrome b* gene. Identification at species level was performed by sequencing analysis. *Plasmodium matutinum* was detected in both dead penguins and in mosquitoes (*Culex pipiens*), while *Plasmodium vaughani* in *Culex pipiens* only. Parasites were not found in any of the PCR tested *Aedes albopictus* samples. Based on our phylogenetic analysis, we detected three previously characterized lineages: *Plasmodium matutinum* LINN1 and AFTRU5, *P. vaughani* SYAT05. In *Culex pipiens* we also identified two novel lineages, CXPIP32 (inferred morphospecies *Plasmodium matutinum*) and CXPIP33 (inferred morphospecies *P. vaughani*). Significantly, LINN1 and AFTRU5 were found to be associated to penguin deaths, although only LINN1 was detected both in penguins (along the years of the study) and in *Culex pipiens*, while AFTRU5 was detected in a single penguin dead in 2017. In conclusion, in our study *Plasmodium matutinum* was found to cause avian malaria in captive penguins kept in Europe, with *Culex pipiens* being its most probable vector. Our results are in agreement with previous studies suggesting that *Culex pipiens* is one of the main vectors of *Plasmodium* spp. in Europe and the Northern Hemisphere. Zoos maintaining captive penguins in temperate areas where *Culex pipiens* is abundant should be well aware of the risks of avian malaria, and should put every effort to prevent outbreaks, in particular during the periods when the number of vectors is higher.

## Introduction

Avian malaria is a parasitic disease of birds caused by protozoa belonging to the genus *Plasmodium*, within the order Haemosporida (1, 2). The genus *Plasmodium* is the largest genus within this order and currently consists of more than 50 species worldwide distributed both in tropical and temperate areas (1–7). Avian malaria identification at species level has traditionally relied on morphological analyses of parasites seen on blood smears. More recently, molecular characterization has led to the description of multiple parasites lineages, and these lineages have been gradually assigned to their respective morphospecies (2, 6, 8, 9). DNA-barcoding of multicellular eukaryotes often target sections of mitochondrial genes, specifically of the *cytochrome c oxidase* subunit I and of the *cytochrome b* (cyt *b*). In particular, the cyt *b* has became the reference gene for DNA-barcoding approaches of avian haemosporidians (2). *Plasmodium* spp. have been reported from almost all avian orders, Galliformes, Columbiformes and Passeriformes being those presenting the greatest parasite diversity (7, 10). In particular, the cosmopolitan *P. relictum* has been recorded in over 400 bird species, belonging to 70 families (3, 7, 10). In Europe, several *Plasmodium* species have been so far detected in both birds and mosquitoes, such as *P. relictum, P. vaughani* (2, 11–15), *P. elongatum* and *P. matutinum* (2, 4, 5, 16, 17). However, as in some studies dealing with avian malaria specific identification of the parasite was not performed, neither morphologically nor through molecular or combined approaches, this list of species should be considered presumably incomplete (18). Indeed, the issue of relating molecular genetic data to classical taxonomy, primarily based on the morphology of blood stages, and the vector and host susceptibility of the parasites, has already been raised (2).

Despite the widespread diffusion of the parasites, avian malaria is not considered a major cause of epidemics or mortalities in natural bird populations that co-evolved with these protozoa (19). Conversely, it is responsible of significant mortalities in captive birds in zoos or collections, when naive bird species are for the first time exposed to the infection, in particular during the spring-summer seasons (1, 3). Avian *Plasmodium* species are transmitted by mosquitoes of many genera within the family Culicidae (10). Different species within the genus *Culex* (*Cx*.) are considered the major vectors in the Northern Hemisphere and in particular *Cx. pipiens* (1, 11, 19–21).

Several species of penguins have been recognized to be highly susceptible to avian malaria in both tropical and temperate areas (1, 6, 22). In particular, the presence of *Plasmodium* infection has been frequently reported both in rehabilitation centers (6, 23–27) and in captive penguin colonies in zoos (9, 17, 28–38), where the disease often caused high morbidity and mortality.

In the Northern Hemisphere and in particular in Europe, the majority of documented avian malaria cases in captive penguins have been caused by *P. relictum* (39–45) and *P. elongatum* (17), although it has to be considered that in some cases identification at species level was not reported (1, 35). As wild birds, in particular passerines, are frequently infected with *Plasmodium* spp. (3), the *Plasmodium* species infecting penguins in a zoo would substantially reflect those present in the local avifauna (27). However, possible differences regarding the virulence of different lineages in different species have been suggested and need to be further investigated (9, 20).

At present, in Italy, information regarding the epidemiology of avian malaria and the impact of the disease in both wild and captive birds is rather few. Some earlier and more recent studies reported the presence of different *Plasmodium* species in wild birds without evidence of disease, including *P. matutinum, P. giovannolai* (16, 46), *P. relictum*, and *P. vaughani* (47), *P. circumflexum* and *P. polare* (48). In another study on blood-fed mosquitoes from northern Italy, Martínez-de la Puente et al. (21) reported the presence of six *Plasmodium* lineages in different mosquito species, including *P. relictum* and *P. vaughani*. In this study, *Cx. pipiens* showed by far the highest parasite prevalence, although one *Plasmodium* lineage was also detected in *Aedes (Ae.) albopicus* (21).

Following the death presumptively attributed to avian malaria in African penguins kept in two zoos located in Central Italy, a multidisciplinary study was carried out in order to describe the pathological findings, to identify and characterize by molecular methods the *Plasmodium* species and lineages responsible for these deaths, and to evaluate the mosquito species possibly involved in the transmission of the disease.

## Materials and Methods

### Studied Populations and Locations

During the summer seasons between 2015 and 2019 the deaths of eight African penguins (*Spheniscus demersus*), tentatively attributed to avian malaria, were recorded among the colonies of two zoos located in Central Italy (Lazio region, Ecoregion: Mediterranean forests, woodlands, and scrub) and situated about 30 km apart, named Bioparco (41.91696 N, 12.48817 E) and Zoomarine (41.63364 N, 12.45661 E). Zoomarine recorded five dead penguins (animal IDs Z1-Z5), while Bioparco three dead penguins (animal IDs B1-B3) (Table 1). These avian malaria deaths accounted for 80% (8/10) of the total penguin deaths at the two colonies during the study period. In all cases, deaths occurred suddenly without any apparent avian malaria-associated clinical sign. Information about zoo of origin, age of the animals and date of deaths of the eight penguins is reported in Table 1.

**Table 1:** Summary of the main metadata (animal ID, age, date of death) of the eight dead penguins examined from both zoos (Bioparco and Zoomarine), and positive for *P. matutinum* by PCR and molecular identification.

**Animal ID**	**Host**	**Zoo of origin**	**Age of the animals (in months)**	**Date of deah**	**Main necropsy findings**	**Main histopathological findings**	**Organs tested by PCR**	**Sequences ID**	**MalAvi lineage**
Z1	*S. demersus*	ZOOMARINE	30	September 2015	Hepato- spleno- nephro- megaly; pale hepatic parenchyma; hydropericardium; encephalic congestion	Periportal lymphoplasmacytic hepatitis; granulomatous splenitis; lymphoplasmacytic pneumonia and hyperemia	Lung, brain	IT01P15	LINN1
Z2	*S. demersus*	ZOOMARINE	8	August 2016	Hepato- spleno- megaly; pale hepatic parenchyma; hydropericardium; severe lung congestion	Periportal lymphocytic hepatitis; interstitial lymphocytic nephritis; lung hyperemia	Liver, lung	IT02P16	LINN1
Z3	*S. demersus*	ZOOMARINE	10	July 2018	Hepato- spleno- nephro- megaly; severe lung congestion; epicardial petechiae	Periportal lymphocytic hepatitis; lymphoplasmacytic pneumonia	Liver, spleen	IT03P18	LINN1
Z4	*S. demersus*	ZOOMARINE	5	July 2019	Hepato- spleno- nephro- megaly; hydropericardium; severe lung edema	Periportal lymphocytic and heterophilic hepatitis; interstitial heterophilic nephritis; multifocal splenic necrosis	Liver, spleen	IT04P19	LINN1
Z5	*S. demersus*	ZOOMARINE	2	July 2019	Hepato- spleno- nephro- megaly; hydropericardium; severe lung edema	Periportal lymphocytic hepatitis; multifocal lymphocytic miocarditis	Liver, spleen	IT05P19	LINN1
B1	*S. demersus*	BIOPARCO	41	July 2017	Hepato- spleno- megaly; severe lung congestion; encephalic congestion	Periportal lymphocytic hepatitis; lymphoplasmacytic enteritis; lymphoplasmacytic pneumonia with hyperemia and multifocal necrosis	Liver, spleen	IT06P17	LINN1
B2	*S. demersus*	BIOPARCO	64	July 2017	Hepato- spleno- megaly; hydropericardium; encephalic congestion; severe lung congestion and edema	Periportal lymphocytic hepatitis; lymphoplasmacytic enteritis; lymphoplasmacytic pneumonia; lymphocytic nephritis	Liver, spleen	IT07P17	AFTRU5
B3	*S. demersus*	BIOPARCO	6	August 2019	Spleno- megaly; pale hepatic parenchyma; hydropericardium	Periportal lymphocytic hepatitis; pulmonary and encephalic hyperemia	Liver, spleen	IT08P19	LINN1

*Main necropsy and histopathological findings are described. SequencesID and MalAvi lineages are also reported*.

The Bioparco is one of the oldest zoological gardens in Europe, founded in 1,911. It is located in the city center of Rome (Italy), covers an area of 18 ha and houses about 1,000 specimens belonging to almost 200 species of mammals, birds and reptiles. Animals live in large spaces with reconstruction of the natural habitats suitable for each species. During the study period the zoo hosted an average population of *S. demersus* of about 20–25 animals.

Zoomarine is a “didactic” theme park (mainly focused on marine life) located in the town of Torvaianica (Rome, Italy) open to the public since 2005. It covers an area of 40 ha and houses about 350 animals of 36 different species (mammals, birds, and reptiles). During the study period the zoo hosted an average population of *S. demersus* of about 15 animals.

In both zoos conventional prophylaxis measures commonly established in zoos to protect the colonies from the risk of avian malaria (22), including vector control and periodic treatment of the animals with antimalarial drugs, were implemented.

During the study period, dead penguins were sent to the Istituto Zooprofilattico Sperimentale del Lazio e della Toscana “M. Aleandri” (IZSLT) to ascertain the causes of death.

### Pathology and Histopathology

On all eight animals a complete necropsy was performed. Samples of multiple organs were collected and fixed in 10% neutral buffered formalin for histopathological examination. For biomolecular analyses, liver and spleen were regularly collected and tested, as they represent the target organs for the diagnosis of avian malaria, but for samples IDs Z1 and Z2 for which brain and lungs were tested instead of the spleen, respectively (Table 1).

Whenever considered pertinent, samples for additional virological and bacteriological analyses were also collected (data not shown).

### Mosquitoes Collection

In the period September-October 2019, mosquitoes samplings were carried out in both zoos where penguin mortalities occurred. Adult mosquitoes were sampled using traps of the model Italian Mosquito Trap (IMT) (a modified CDC light trap with an insulated bucket holding the dry ice, hence the CO_2_ is dispensed from above) lured with 1 kg of dry ice (CO_2_) and Gravid Traps lured with a mixture of water and hay soiled with guinea pig feces and urine. Traps were located as close as possible to the penguin enclosures. For each sampling session, three IMT traps and one Gravid trap run from late afternoon to 8:30–10:00 of the following morning. A total of five sampling sessions at Bioparco and six at Zoomarine were performed. Noteworthy, at Bioparco, in order to try to protect penguins from mosquitoes, the animal health managers had placed in their enclosure two traps model BG-Mosquitaire (Biogents) baited with CO_2_ supplied with a gas cylinder and continuously operating. During each sampling session, mosquitoes caught by these traps were also collected.

Considering the different traps used during each sampling session, 21 and 18 catches were performed at Bioparco and Zoomarine, respectively, comprehensive of the collections of the two BG-Mosquitaire.

Mosquitoes were morphologically identified at the laboratory of Parasitology and Entomology- General Diagnostic Department of IZSLT, following the identification keys by Severini et al. (49). During the sorting and identification process, all mosquitoes were checked for the presence of blood in the abdomen. After identification, whole mosquito females were pooled in groups of about 30–50 specimens (for a total of 38 pools) and transferred for molecular analyses (Table 2).

**Table 2:** Number of mosquitoes caught/identified at both zoos (Bioparco and Zoomarine) and tested in pools by PCR for the presence of *Plasmodium* spp.

	***Culex pipiens***				***Aedes albopictus***		
**Zoo of origin**	**Caught/identified**	**PCR tested (females only)**	**PCR positive (cyt *b* gene)**	***Plasmodium* Species identified/lineage**	**Caught/identified**	**PCR tested (females only)**	**PCR positive (cyt *b* gene)**
Bioparco	1,973	1,888 (19 pools)	1 pool	*P. matutinum*/LINN1	1,074	515 (10 pools)	None
Zoomarine	265	265 (8 pools)	3 pools	*P. matutinum* (1 positive pool)/CXPIP32^*^	62	30 (1 pool)	None
				*P. vaughani* (2 positive pools)/CXPIP33^*^-SYAT05			

* *Inferred morphospecies*.

### *Plasmodium* Molecular Identification and Characterization

Total DNA was extracted from the above-mentioned penguin organs and from pooled mosquitoes. Before extraction, the mosquito pools were placed in tubes containing beads and mechanically homogenized using the Tissuelyser (Qiagen) at 30 Hz for 3 min, followed by a centrifugation at 17 g for 10 min.

From both organs and mosquitoes pools, DNA extraction was performed using an automated system (QIAsymphony SP Qiagen) with the DSP Virus/Pathogen Mini Kit, following the manufacturer's instructions.

Extracted DNA was subjected to a simplex PCR assay for the detection of the three haemosporidian genera (*Plasmodium, Haemoproteus* and *Leucocytozoon*), using as a target the partial mitochondrial conserved region of the *cytochrome b* gene (cyt *b*). The PCR was performed with the primers HaemNFI (5′-catatattaagagaaitatggag-3′) and HaemNR3 (5′-atagaaagataagaaataccattc-3′) described by Hellgren et al. (50), using the following protocol: Master Mix was prepared with 5 μL of distilled water, 12.5 μL of Platinum™ Hot Start PCR Master Mix 2X (Invitrogen), 1.25 μL of each M13-tailed primer (10 pMol/ μL), with a final volume of 20 μL, then 5 μL of the template was added.

PCR started with an initial denaturation step of 3 min at 95°C, followed by 35 cycles of 15 s at 95°C, 15 s at 50°C, 5 s at 72°C, and a final extension at 72°C for 3 min. Negative and positive controls for both DNA extraction and PCR reaction processes were also included. For sequencing, positive PCR-products (570 bp) were purified using an enzymatic cleanup ExoSAP-IT™ kit. Amplicons were Sanger sequenced on a 3,500 Series Genetic Analyzer with BigDye Terminator chemistry (Applied Biosystems, USA) using the same primers. Sequence data analysis and trimming was performed using the CLC DNA workbench® software version 5.7.1. The resulting sequences were examined for the presence of overlapping peaks to exclude potential co-infections and compared using BLAST (online version^1^, blastn algorithm) (51). The cyt *b* sequences were also compared for similarity to sequences available at the MalAvi database^2^.

For further characterization, the multiple sequence alignment (MSA) of the Sanger sequences was performed by using MUSCLE algorithm with default settings (53).

For the phylogenetic analysis, all the cyt *b* sequences obtained from mosquito pools were included, while to avoid clutter of the tree, only one cyt *b* sequence (when identical among organs) from each dead penguin was included. In addition, three *Plasmodium* sequences publicly available (54) were used as references (*P. matutinum* AFTRU5, GenBank MK652236; *P. matutinum* LINN1, GenBank MK652235; *P. vaughani* SYAT05, GenBank MK652243). All *Plasmodium* cyt *b* sequences previously reported in the phylogenetic tree of Valkiūnas et al. (5), except for *P. juxtanucleare* GALLUS03, which showed low quality, were also included. Finally, *P. relictum* ATCC 30141, GenBank AY099032.1 and *P. lutzi* TFUS05 (6) sequences were included. *Leucocytozoon schoutedeni* GALLUS06 was used as outgroup.

The length of the final alignment used to build the phylogenetic tree was 454 bp This length refers to the smallest cyt *b* sequence obtained following alignment. The alignment file and the p-distance matrix calculated with R are provided as Supplementary Data Sheets 1, 2, respectively.

All the sequences obtained in this study and used for the phylogenetic analyses were submitted to the European Nucleotide Archive (ENA)^3^ under the study accession number PRJEB41796.

A comparative genomics analysis of the MSA, with Bayesian inference of phylogeny, was performed by using MrBayes version 3.2 (55). The General Time Reversible Model including invariable sites and variation among sites (GTR + I + G) was used. Two independent runs were performed. Each analysis was run for a total of five million generations with a sampling frequency of every 100 generations. Before constructing a majority-rule consensus tree, 25% of the initial trees in each run were discarded as “burn in” periods.

## Results

### Pathology and Histopathology

At necropsy, gross lesions were almost similar in all the eight penguins examined, regardless of the origin (Bioparco or Zoomarine). The livers were generally moderately megalic, with pale and degenerated parenchyma, and the spleen were severely enlarged, in some cases up to six times the normal size. Lungs were severely affected, either congested or oedematous, or both. In almost all cases (7 out of 8), a severe serous pericardial effusion was observed. Other remarkable lesions were hyperaemia of the intestinal serosa (4/8), red discolouration of the cerebral parenchyma (3/8) and nephromegaly (3/8). In one case, multiple white thickened plaques were observed on the air sacs.

Inflammatory and degenerative lesions were microscopically observed in multiple organs, in particular in the liver, lungs and brain. A moderate to severe lymphocytic periportal hepatitis was regularly observed, with pronounced haemosiderosis and multifocal necrosis in few cases. Lungs were diffusely affected, severely congested with multifocal hemorrhages and a mild to moderate inflammatory lymphocytic infiltrate; small multifocal foci of necrosis were observed in three cases. In the spleen, a moderate lymphoreticular hyperplasia was evident in all cases and necrosis was observed in two animals. In the brain, the most evident changes were congestion and perivascular oedema. In all the examined organs, numerous 20 to 80 μm in diameter round to elongate structures (schizonts), filled with uncountable 1−3 μm in size intensely basophilic nuclei (merozoites), were observed inside the cytoplasm of endothelial or reticuloendothelial cells (intracellular exoerythrocytic stage), suggestive of the presence of *Plasmodium* spp. (Supplementary Figure 1); the resulting obstruction of the lumen of the capillaries was particularly evident in brain, kidney and the lung tissue sections. A summary of the main necropsy and histopathological findings are reported in Table 1.

Gross and histopathological lesions, together with the presence of merozoites and absence of other evident causes of death (data not shown), confirmed the diagnosis of avian malaria.

### Mosquitoes Collection

In both zoos, *Cx. pipiens* was the dominant species (1,973 specimens at Bioparco, 265 at Zoomarine), followed by *Ae. albopictus* (1,074 specimens at Bioparco, 62 at Zoomarine). Other minor species identified were *Culiseta longiareolata* at Bioparco (five specimens), and *Ae. vexans* at Zoomarine (six specimens). These latter minority species were not tested by PCR for the presence of Haemosporida.

As expected, the working continuously BG-Mosquitaire traps gave the highest catch rate.

The number of mosquitoes identified, as well as the number of female mosquitoes tested (in pool) by PCR for the presence of avian haemosporidian protozoa (*Plasmodium, Haemoproteus* and *Leucocytozoon*) are reported in Table 2. None of the PCR tested mosquitoes resulted even partially engorged of blood in the abdomen. Detailed information about the number of mosquitoes of each species collected in each sampling session (for each type of trap), is reported in Supplementary Table 1.

### *Plasmodium* Identification and Characterization

All the analyzed organs (*n* = 16) of the eight dead penguins from both zoos tested PCR positive (Supplementary Figure 2). Blast analysis demonstrated a nucleotide identity with *P. matutinum* for all the obtained penguin sequences. As for the mosquito pools, four *Cx. pipiens* pools tested PCR positive, while the *Ae. albopictus* pools tested all negative. Sequence analysis of *Cx. pipiens* pools demonstrated the presence of two *Plasmodium* species detected at Zoomarine, namely *P. vaughani* (two positive pools) and *P. matutinum* (one positive pool), while at Bioparco only *P. matutinum* was detected (Table 2). Assuming that in each positive pool just one mosquito was positive for *Plasmodium* spp., the estimated minimum infection rate (MIR) at Bioparco was 0.05% (1/1,888), while at Zoomarine 1.13% (3/265). Specifically at Zoomarine, *P. vaughani* MIR was 0.75% (2/265), while *P. matutinum* MIR was 0.38% (1/265).

Analyzing the chromatograms, no evidence of double peaks (suggestive of mixed infections) was detected in any of the sequences. Overall, eight *P. matutinum* sequences selected from the dead penguins (one for each penguin), and all the four sequences from the positive *Cx. pipiens* pools (two identified as *P. matutinum* and two as *P. vaughani*) were included in the phylogenetic analysis.

**Figure 1:**
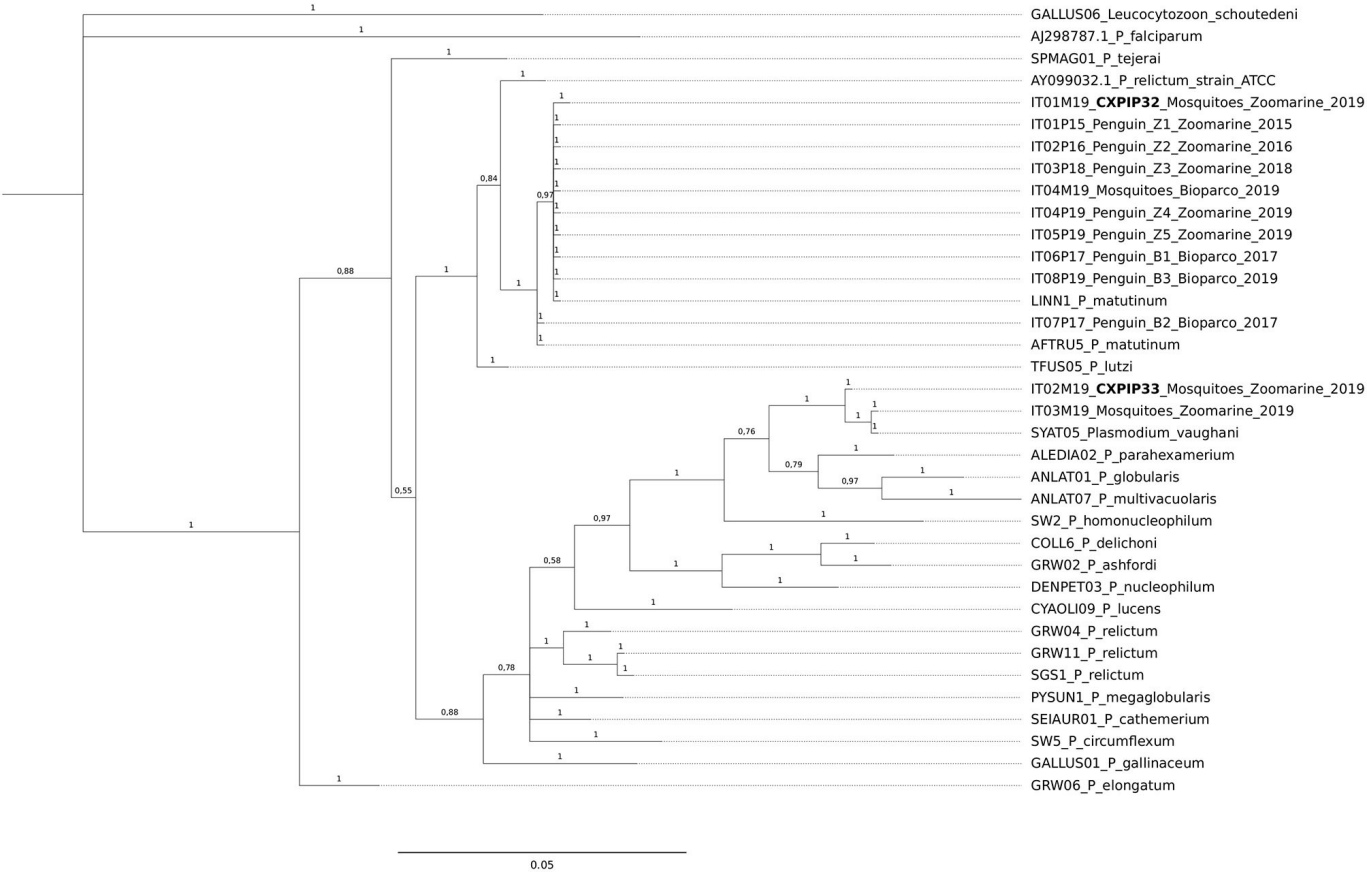
Bayesian tree of *n* = 36 mitochondrial cyt *b* sequences of *Plasmodium* species, including 12 *Plasmodium* sequences obtained in this study from penguins and from mosquito pools, 23 reference lineages from MalAvi database and one ATCC from GenBank. Branch lengths are drawn proportionally to the extent of changes (scale bar is shown). Values adjacent to nodes represent posterior probabilities. IDs of the sequences obtained from penguins and mosquito pools in relation to the animal IDs, location and year of death/trapping, are reported. In bold the two newly identified MalAvi lineages.

From the obtained tree it can be inferred that all but one of the *P. matutinum* sequences detected in this study were identical to two previously characterized and closely related lineages (LINN1 and AFTRU5, Figure 1). In a *Cx. pipiens* pool collected at Zoomarine (ID IT01M19) we also identified one novel lineage, not 100% identical to LINN1 (p-distance = 0.0022, Supplementary Data Sheet 2). LINN1 and AFTRU5 were found to be associated to penguin deaths, although only LINN1 was detected both in penguins (along the years of the study) and in *Cx. pipiens*, while AFTRU5 was detected in a single penguin dead in 2017 (ID IT07P17). One of the two *P. vaughani* sequences (IT03M19) obtained from *Cx. pipiens* pools collected at Zoomarine was identical to a previously characterized lineage (SYAT05), while the other (ID IT02M19), was slightly different (p-distance = 0.0044, Supplementary Data Sheet 2).

Sequences IT01M19 and IT02M19 obtained from *Cx. pipiens* were assigned the MalAvi lineage names CXPIP32 and CXPIP33, respectively; phylogenetic analysis suggests they correspond to *P. matutinum* and *P. vaughani*, respectively.

## Discussion

We used a multidisciplinary approach to investigate the death due to avian malaria in eight African penguins (*S. demersus*) kept in two Italian zoos, thus providing new information regarding the pathology, molecular identification and phylogenetic characterization of a *Plasmodium* (*P. matutinum*) causing infection in these captive birds. We also provided information about the presence and circulation of avian *Plasmodium* species in mosquitoes from Central Italy.

Gross pathological and histopathological findings detected in the dead penguins were consistent with those previously observed by other authors (22, 26, 29, 37, 38). Lesions in all the animals were acute to peracute, with massive diffusion of the parasites in tissues macrophages, mainly in liver and spleen and representing an exoerythrocytic pathway of infection, with endothelial involvement in more advanced cases. This pathway has already been described and in penguins it is considered the most frequent way of replication of *Plasmodium* spp. (1, 29, 37, 38).

Regarding the vectors, our results are in agreement with previous studies and provide strong evidence that *Cx. pipiens* is one of the main vectors of *Plasmodium* spp. associated with avian malaria in Europe and the Northern Hemisphere (12, 15, 20). Indeed, this species has been already reported as responsible for avian malaria transmission to captive penguins in zoos (1, 15, 56). Importantly, in our study avian haemosporidia were not found in *Ae. albopictus*. The role of this latter species as possible vector of avian malaria is quite controversial. Some authors reported the detection of these parasites in *Ae. albopictus*, even though with low prevalence values (21, 57), some others reported the absence of *Plasmodium* spp. in this mosquito species (58, 59). Beyond these findings, it is important to remind that *Ae. albopictus* has a marked preference for mammals (15, 60, 61), hence it is unlikely that this species will play an epidemiologically relevant role in the transmission of avian malaria.

Regarding the *Plasmodium* spp. MIR values in *Cx. pipiens*, when compared to data reported in other studies, our estimates appear quite low. In fact, the overall MIR at Zoomarine was 1.13%, similar to the total MIR (0.99%) reported in the same mosquito species by Schoener et al. (4) in a three-year study in Eastern Austria, but significantly lower than the maximum MIR (5.26%). Even more marked are the differences when comparing our findings with those described by Lalubin et al. (20) and Martínez-de la Puente et al. (21) reporting prevalence values of 15.4% and 30% in *Cx. pipiens* in Switzerland and North-Eastern Italy, respectively. Our results are of some concern, since even with a very low MIR, it seems that both transmission and disease can occur causing mortality in captive penguins. Nevertheless, it should be taken into account that our investigation on mosquitoes was not specifically aimed at the definition of prevalence estimates, and these results need to be confirmed and further investigated.

Regarding the higher number of dead penguins and positive mosquito pools recorded at Zoomarine compared to Bioparco, we cannot rule out that differences of the habitat, wild bird community and/or management (e.g., measures put in place to limit mosquito exposure), might have played an important epidemiological role. The greater habitat heterogeneity in the surroundings of Zoomarine (e.g., cultivated fields, streams, proximity to sea and to two Natural Parks) may have positively influenced key community parameters (e.g. species richness, abundance and distribution), increasing the frequency of vector-host contacts (62, 63). Indeed, the two sites significantly differed in environmental features related with suitable mosquito breeding habitats. For instance, a stream in the proximity of the penguin enclosure at Zoomarine, where most of the mosquito collections were performed, has been recognized as the most probable larval development site. Nevertheless, being the stream under public ownership, the water could not be treated with larvicids, as instead it is usually done at Bioparco for standing waters close to the enclosures. Given the tendency of *Cx. pipiens* to feed on avian hosts (64), the water stream, providing habitat for both vectors and birds, may have increased the frequency of reservoir-vector-penguin contacts.

On the genomic side, we detected three previously characterized lineages (LINN1, AFTRU5, SYAT05) and identified two novel lineages in mosquitoes, CXPIP32 (inferred morphospecies *P. matutinum*) and CXPIP33 (inferred morphospecies *P. vaughani*). Interestingly, LINN1 and AFTRU5 were found to be associated to penguin deaths, although only LINN1 was detected both in penguins (along the years of the study, in both zoos) and in *Cx. pipiens*, while AFTRU5 was detected in a single penguin dead in 2017 at Zoomarine.

On the other hand, SYAT05, CXPIP32, and CXPIP33 were detected only in *Cx. pipiens*. SYAT05 is considered a common generalist avian malaria parasite (2, 7), but so far in Italy it has been previously detected only in *Cx. pipiens* samples collected in the north-east (21) and in skylark (*Alauda arvensis*) in southern Italy (47).

*P. matutinum* and related lineages have already been associated with mortality in penguins and other birds. Sijbranda et al. (37) analyzing archived tissues using a nested PCR for *Plasmodium* spp. followed by DNA sequencing, revealed that two wild little penguins (*Eudyptula minor*) found dead on New Zealand beaches were infected with lineage LINN1. Vanstreels et al. (6) investigating an outbreak of avian malaria in Magellanic penguins (*Spheniscus magellanicus*) at a rehabilitation center in southeast Brazil identified a *Plasmodium* sp. PHPAT01 infection in a penguin with post-mortem lesions suggestive of avian malaria. This lineage was already determined to have caused the death of a Magellanic penguin in a previous study (27). The morphospecies of PHPAT01 has yet to be determined, but the authors suggest it is most closely related to *P. lutzi* and *P. matutinum* (6). Dinhopl et al. (65) reported that partial cyt *b* sequences deposited as lineages AFTRU5 or LINN1 and tentatively attributed to the species *P. lutzi*, showed homology to two previously obtained sequences from two captive penguins kept in the Vienna zoo. Finally Spottiswoode et al. (9), in a study conducted in captive penguins (Sphenisciformes spp.), eiders (*Somateria* spp.), and inca terns (*Larosterna inca*) in a North American zoological collection, reported *P. matutinum* LINN1 in several necropsied terns, but only in a healthy penguin subjected to surveillance. Our results would confirm the pathogenicity of *P. matutinum* (LINN1 and AFTRU5) in penguins; interestingly we detected this same species causing mortalities in two different zoos situated about 30 km apart and through several years. To note that Corradetti et al. (66) already reported the presence of *P. matutinum* in a wild bird in Italy (redwing, *Turdus iliacus*), although in this case the identification at species level was only phenotypic (microscopic examination). *P. matutinum* has also been previously reported as most closely related to *P. tejerai* (cyt *b* lineage SPMAG01), a common malaria parasite of American birds, based on partial cyt *b* sequences and on phenotypic characteristics (5). Indeed, *P. tejerai* is considered highly lethal for *Spheniscus magellanicus*, a species closely related to African penguin, with 75% (12/16) case-fatality rate among individuals found infected at rehabilitation centers in Brazil (27).

Avian malaria is a well-known and relevant problem for penguins kept at zoos or rehabilitation centers worldwide (1, 22, 26, 27). The susceptibility to the disease caused by *Plasmodium* in penguin species is usually interpreted in the light of a poor parasite adaptation of the hosts, due to their naïve status in their original area of distribution (19, 22). In these situations, without any kind of prophylactic and protective measure, there is a high risk of registering continuous clinical cases and deaths in colonies. In this scenario, both at Bioparco and Zoomarine, preventive measures are adopted, in particular during the seasons when adult mosquitoes are active. Specifically, at Bioparco, strategies aiming at reducing the number of mosquitoes around the penguins using different approaches are adopted. These strategies are: the use of traps (BG-Mosquitaire), the use of powerful fans able to create a strong air flow in the area of the enclosure where animals use to spend the night, the use of water vents in areas not reached by the air fans, the spraying of the nests surroundings with neem oil and the treating of standing waters with larvicides (67). Furthermore, both at Bioparco and Zoomarine, according to renowned protocols (22), penguins are periodically treated with chloroquine phosphate and at Zoomarine also with primaquine. These measures surely limited the impact of avian malaria on the two colonies, in particular at Bioparco, even though they were not sufficient to fully prevent the occurrence of clinical cases and deaths.

Avian malaria, in Italy, is poorly known and investigated. Very few studies in the past focused on haemosporidian infections, due to the perceived limited clinical relevance of these parasites, usually quietly coexisting with their natural hosts. Indeed, silent or subclinical infections are frequent in many avian vertebrate hosts, possibly imposing fitness costs based on trade-off mechanisms, ranging from decreased survival to decreased fecundity in certain species (68). The presence of two colonies of African penguin brought to the light something that otherwise could have gone unnoticed. Interestingly, the deaths occurred in 2017 in the penguin colony of the Bioparco of Rome (n = 2 animals, IDs B1 and B2, Table 1) occurred immediately after the translocation of these animals from a zoo located in Northern Italy. The B1 subject was also the only dead penguin in which a *P. matutinum* AFTRU5 lineage was detected, a lineage not found in the collected mosquitoes. Remarkably, AFTRU05 has been already recorded in mosquitoes in northern Italy (21). Hence, it cannot be ruled out that the two birds, or at least B1, arrived in Rome already infected, and that the stress of the translocation might have favored a reactivation of the disease. Indeed, this kind of stress is a well-known risk factor for developing clinical signs of avian malaria in captive birds (22) and should be carefully considered.

Zoos maintaining captive penguins in temperate areas where *Cx. pipiens* is abundant should be well aware of the risks of avian malaria, and should put every effort to prevent outbreaks, in particular during the periods when the number of vectors is higher.

## Data Availability Statement

The datasets presented in this study can be found in online repositories. The names of the repository/repositories and accession number(s) can be found in the article/Supplementary Material.

## Author Contributions

MI, FR, CD, CC, KF, FM, and AB: conceived and designed the experiments. MI, FR, CC, FS, AM, RO, and AB: performed the experiments. ED, MI, PA, VC, AC, CC, CD, and AB: analyzed the data. MI, CC, ED, FR, VC, AC, CD, and AB: wrote the paper.

## Conflict of Interest

The authors declare that the research was conducted in the absence of any commercial or financial relationships that could be construed as a potential conflict of interest.

## Footnotes

^1^https://blast.ncbi.nlm.nih.gov/Blast.cgi

^2^http://mbio-serv2.mbioekol.lu.se/Malavi (52)

^3^http://www.ebi.ac.uk/ena

## Supplementary Material

Supplementary Figure 1 Histopathological pictures (Hematoxylin-eosin). **(a)** *Spheniscus demersus*, liver. Moderate lymphocytic periportal hepatitis with intralesional merozoites (arrows). **(b)** *Spheniscus demersus*, brain. Intra-endothelial merozoites obstructing the lumen of the brain capillary (arrow).

Supplementary Figure 2 Gel image of the PCR results of the cyt *b* target obtained from the automated capillary electrophoresis high-resolution system QIAxcel System® (Qiagen), from left to right: Lane 1: Size Marker (*QX* DNA *Size Marker* 100 bp−2.5 kb). Lanes 2-17: cyt *b* amplicon from dead penguins (lane 11, weak positive sample). Lanes 18-21: cyt *b* amplicon from mosquito pools. Lane 22: Negative control (Nuclease-Free Water). Lane 23: Positive control (*Plasmodium spp*. DNA, IZSLT collection).

Supplementary Data Sheet 1 Alignment of the 454 bp cyt *b* sequences used to build the phylogenetic tree.

Supplementary Data Sheet 2 p-distance matrix of the 454 bp cyt *b* sequences used to build the phylogenetic tree, calculated with R.

Supplementary Table 1 Detailed information about the number of mosquitoes of each species collected in each sampling session, for each type of trap, with the results of molecular identification and MalAvi lineage.
