# Supplementary figures and images for: Plasmodium matutinum Transmitted by Culex pipiens as a Cause of Avian Malaria in Captive African Penguins (Spheniscus demersus) in Italy
Source: Front Vet Sci. 2021 Mar 16;8:621974. doi: 10.3389/fvets.2021.621974 (PMC8009178; doi:10.3389/fvets.2021.621974)

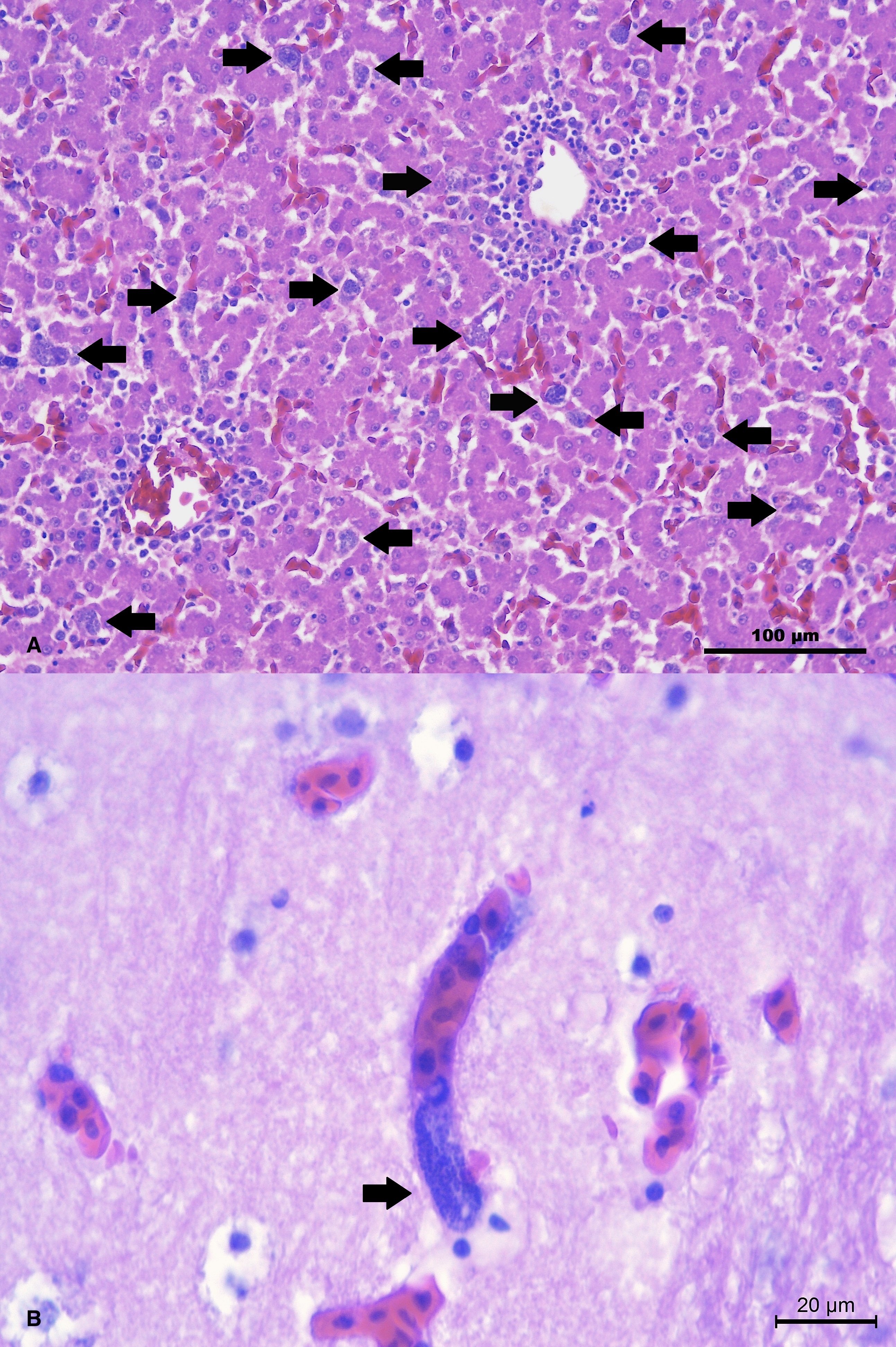

Supplement: Supplementary Figure 1 — Histopathological pictures (Hematoxylin-eosin). (a) Spheniscus demersus, liver. Moderate lymphocytic periportal hepatitis with intralesional merozoites (arrows). (b) Spheniscus demersus, brain. Intra-endothelial merozoites obstructing the lumen of the brain capillary (arrow). [file Image_1.JPEG]

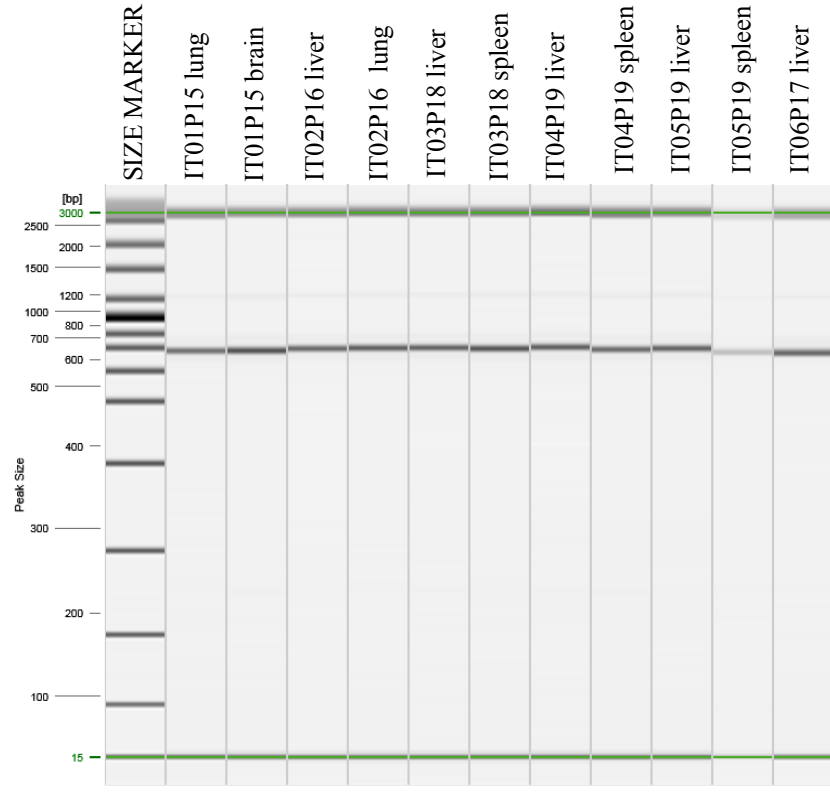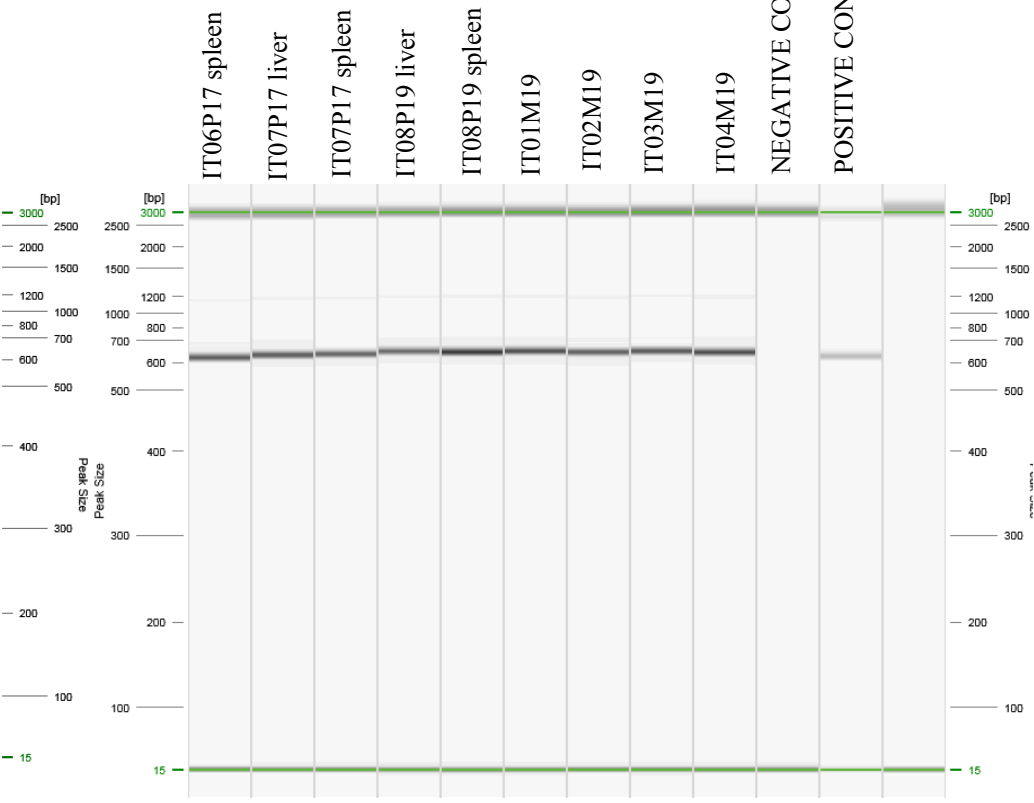

Supplement: Supplementary Figure 2 — Gel image of the PCR results of the cyt b target obtained from the automated capillary electrophoresis high-resolution system QIAxcel System® (Qiagen), from left to right: Lane 1: Size Marker (QX DNA Size Marker 100 bp−2.5 kb). Lanes 2-17: cyt b amplicon from dead penguins (lane 11, weak positive sample). Lanes 18-21: cyt b amplicon from mosquito pools. Lane 22: Negative control (Nuclease-Free Water). Lane 23: Positive control (Plasmodium spp. DNA, IZSLT collection). [file Image_2.PDF]
