# Supplementary material for: Plasmodium matutinum Transmitted by Culex pipiens as a Cause of Avian Malaria in Captive African Penguins (Spheniscus demersus) in Italy
Source: Front Vet Sci. 2021 Mar 16;8:621974. doi: 10.3389/fvets.2021.621974 (PMC8009178; doi:10.3389/fvets.2021.621974)
Supplement: Supplementary Table 1 — Detailed information about the number of mosquitoes of each species collected in each sampling session, for each type of trap, with the results of molecular identification and MalAvi lineage. [file Table_1.docx]

**Supplementary Table 1:** Detailed information about the number of mosquitoes of each species collected in each sampling session, for each type of trap, with the results of molecular identification andMalAvi lineage.

| **a) Zoo of origin:**  **Zoomarine** | **Mosquito species** | | | | | | **Σ *Cx. pipiens*** | ***Plasmodium* species Sequences ID** | **MalAvi lineage** |
| --- | --- | --- | --- | --- | --- | --- | --- | --- | --- |
|  | *Cx. pipiens* | | *Ae. albopictus* | | *Ae. vexans* | |  |  |  |
| **Date of sampling** | **Type of trap** | | | | | |  |  |  |
|  | IMT | Gravid | IMT | Gravid | IMT | Gravid |  |  |  |
| 06/09/19 | 9 | 49 | 2 | 3 | 0 | 0 | 58 | *P.matutinum*/  IT01M19 | CXPIP32* |
|  |  |  |  |  |  |  |  | *P.vaughani*/ IT02M19 | CXPIP33* |
| 13/09/19 | 24 | 118 | 4 | 2 | 0 | 0 | 142 | *P.vaughani*/ IT03M19 | SYAT05 |
| 20/09/19 | 7 | 24 | 7 | 1 | 0 | 0 | 31 | Negative | - |
| 27/09/19 | 4 | 9 | 3 | 2 | 0 | 0 | 13 | Negative | - |
| 04/10/19 | 7 | 1 | 0 | 3 | 0 | 0 | 8 | Negative | - |
| 11/10/19 | 8 | 1 | 13 | 4 | 6 | 0 | 9 | Negative | - |
| 18/10/19 | 1 | 3 | 2 | 16 | 0 | 0 | 4 | Negative | - |

* inferred morphospecies

| **b) Zoo of origin:**  **Bioparco** | **Mosquito species** | | | | | | | | | **Σ *Cx. pipiens*** | ***Plasmodium* species/Sequences ID** | **MalAvi lineage** |
| --- | --- | --- | --- | --- | --- | --- | --- | --- | --- | --- | --- | --- |
|  | *Cx. pipiens* | | | *Ae. albopictus* | | | *Cs. longiareolata* | | |  |  |  |
| **Date of sampling** | **Type of trap** | | | | | | | | |  |  |  |
|  | IMT | BG-Mosquitaire | Gravid | IMT | BG-Mosquitaire | Gravid | IMT | BG-Mosquitaire | Gravid |  |  |  |
| 05/09/19 | 16 | 1502 | 13 | 0 | 548 | 7 | 0 | 0 | 2 | 1531 | *P.matutinum* IT04M19 | LINN1 |
| 26/09/19 | 3 | 159 | 14 | 2 | 257 | 0 | 0 | 0 | 0 | 176 | Negative | - |
| 02/10/19 | 2 | 54 | 8 | 1 | 73 | 1 | 0 | 0 | 0 | 64 | Negative | - |
| 10/10/19 | 4 | 39 | 1 | 59 | 0 | 5 | 0 | 0 | 0 | 44 | Negative | - |
| 17/10/19 | 3 | 48 | 3 | 0 | 52 | 5 | 0 | 1 | 0 | 54 | Negative | - |
| 23/10/19 | - | 39 | - | - | 37 | - | - | 2 | - | 39 | Negative | - |
| 30/10/19 | - | 65 | - | - | 27 | - | - | 0 | - | 65 | Negative | - |
